# Efficacy of intralesional meglumine antimoniate in the treatment of canine tegumentary leishmaniasis: A Randomized controlled trial

**DOI:** 10.1371/journal.pntd.0011064

**Published:** 2023-02-15

**Authors:** Jamile Lago, Deborah Fraga, Luiz Henrique Guimarães, Tainã Lago, Yuri Santos, Ednaldo Lago, Guilherme L. Werneck, Olívia Bacellar, Edgar M. Carvalho

**Affiliations:** 1 Immunology Service, Professor Edgard Santos University Hospital Complex, Federal University of Bahia, Salvador, Bahia, Brazil; 2 Post-Graduate Course in Health Sciences, Federal University of Bahia Medical School. Salvador, Bahia, Brazil; 3 Gonçalo Moniz Institute (IGM), Fiocruz, Salvador, Bahia, Brazil; 4 Federal University of Southern Bahia, Teixeira de Freitas, Bahia, Brazil; 5 Instituto Nacional de Ciência e Tecnologia em Doenças Tropicais (INCT-DT), Ministério da Ciência e Tecnologia e Inovação (MCTI), CNPq, Salvador, Bahia, Brazil; 6 Department of Epidemiology, State University of Rio de Janeiro, Rio de Janeiro, Brazil; 7 Institute for Public Health Studies, Federal University of Rio de Janeiro. Federal; Oswaldo Cruz Institute, BRAZIL

## Abstract

Dogs living in areas of *Leishmania (Viannia) braziliensis* transmission may present canine tegumentary leishmaniasis (CTL) characterized by cutaneous or muzzle ulcers as well as asymptomatic *L*. *braziliensis* infection. It is not clear if dogs participate in the transmission chain of *L*. *braziliensis* to humans. However, dogs may remain with chronic ulcers for a long time, and as there are no public policies about CTL, these animals die or are sacrificed. Here we compare the efficacy of intralesional meglumine antimoniate with intralesional 0.9% NaCl solution in CTL treatment. This randomized control study included 32 dogs with cutaneous or muzzle lesions who had *L*. *braziliensis* DNA detected by PCR in tissue biopsied. Group one received 5ml of intralesional Glucantime, and group two received 5ml 0.9% NaCl solution, both applied in the four cardinal points on days 0, 15, and 30. Cure was defined as complete healing of the ulcers in the absence of raised borders on day 90. There was no difference in animals’ demographic and clinical features in the two groups (p >.05). While at the endpoint, the cure rate was 87.5% in the group test, and in those who received 0.9 NaCl the cure rate was only 12.5%. As important as the high cure rate, the healing time was faster in dogs treated with antimony than in those treated with saline (p < .001). Intralesional meglumine antimoniate is effective in the treatment of dogs with *L*. *braziliensis* infection and accelerates the healing time of CTL.

## Author summary

Dogs play an important role in the transmission of *Leishmania infantum*, the causal agent of visceral leishmaniasis. But very little is known about *L*. *braziliensis* infection in dogs. Canine tegumentary leishmaniasis (CTL) caused by *L*. *braziliensis* is documented in endemic areas of human cutaneous leishmaniasis. CTL occurs predominantly in males, and the scrotal sac and muzzle are the primary site of infection. As there are no policies regarding therapy for CTL, dogs die from the disease or are sacrificed. A few uncontrolled studies showed a cure of CTL after treatment with intralesional Glucantime (Sanofi-Aventis). Here, we compare the efficacy of intralesional Glucantime (N = 16) versus intralesional saline (N = 16) in the treatment of CTL. All dogs presented ulcerated lesions. The DNA of *L*. *braziliensis* was documented by PCR in biopsied tissue of all animals in the study. There were no differences among the two groups regarding age, gender, illness duration, lesion size, and lesion site. However, a higher cure rate (87.5%) was observed in the dogs treated with Glucantime compared to 12.5% in those treated with saline (p < .001). This study showed that intralesional Glucantime cures dogs infected with *L*. *braziliensis* and should be used to treat canine tegumentary leishmaniasis.

## Introduction

*Leishmania (Viannia) braziliensis* is the main causal agent of American tegumentary leishmaniasis (ATL), a disease with different clinical forms including cutaneous leishmaniasis (CL), mucosal leishmaniasis (ML) and disseminated leishmaniasis (DL) [1–3]. *Nyssomia intermedia*, *N*. *Whitmani* and *Lutzomyia migonei* are the main vectors of *L*. *braziliensis*. Brazil is the main country where *L*. *braziliensis* transmission is observed and the village of Corte de Pedra, located in the southeast region of the state of Bahia, is one of the most important endemic areas of ATL in Latin America. In this village, a reference center for diagnosis and treatment of tegumentary leishmaniasis, also known as the Corte de Pedra Health Post was built in 1986. In the last four years, 6740 new cases of the disease have been diagnosed.

The prevalence of *L*. *braziliensis* in the canine population of Corte de Pedra has not been determined. However, dogs are found infected with this species of *Leishmania* and canine tegumentary leishmaniasis (CTL) has been observed in this endemic area [4]. In a survey of 413 dogs living about 200km north of the reference center, the polymerase chain reaction (PCR) for *L*. *braziliensis* in skin biopsy tissue of healthy dogs was positive in 226 (54.7%), and 32 (7.7%) of the animals had CTL, indicating a ratio of 7:1 from infection to disease [5]. In another study in the southern region of Brazil, parasites were isolated from ulcers in six (75%) of eight dogs with typical CL skin lesions, and all parasites were identified as *L*. *braziliensis*. [6]. Recently, visiting subjects living around the Corte de Pedra Health Post that had a recent or previous diagnosis of CL, we identified 61 dogs with cutaneous ulcers indicative of CL. The DNA of *L*. *braziliensis* was detected by PCR in 41 (61%) of the animals. CL was present in 35 (85%) of them, ML in five (12%), and one (3%) had both cutaneous and mucosal disease [4]. The age of the dogs ranged from three to five years; 36 (88%) were male. The delayed-type hypersensitivity leishmania skin test was positive in 32 (78%), and anti *L*. *braziliensis* IgG antibodies were detected in 37 (90%) of the animals [4].

Dogs may also be infected with other leishmania species, and *Leishmania infantum*, the causal agent of visceral leishmaniasis (VL), is frequently documented in asymptomatic and symptomatic dogs in VL endemic areas. Dogs have been recognized to play an important role in *L*. *infantum* transmission to humans [7,8]. Because they do not respond to treatment with meglumine antimoniate, the drug recommended to treat human leishmaniasis in Latin America, the recommendation of the Brazilian Government is to perform serological tests to detect antibodies to *L*. *infantum* and to sacrifice *L*. *infantum* infected dogs [9–11].

There are no public policies regarding CTL. Dogs with symptoms of leishmaniasis die or are sacrificed. The literature is scarce on the treatment of CTL in dogs, but the treatment of ATL caused by *L*. *braziliensis* in humans is a challenge. Meglumine antimoniate is used with a dose of 20mg/Kg/weight and a maximum daily dose of 1200mg applied intravenously or intramuscular for 20 days. However, as cutaneous ulcers take a long time to heal, failure or cure is determined only 90 days after therapy starts. The cure rate of ATL in our endemic area was 90% in early 1990, but has decreased over the years. In the last 10 years, cure has been observed in only 40 to 60% of the treated patients [12–15]. A second course of meglumine antimoniate in the same dose for 30 days is prescribed in case of failure. Patients who fail to heal with a second course of this drug are treated with deoxycholate or liposomal amphotericin B. More recently, we showed that the cure rate of CL with miltefosine is higher than with meglumine antimoniate [15] and the Brazilian Government has recommended and provided miltefosine for patients with severe disease who fail to respond to antimony [15,16]. Intralesional meglumine antimoniate was used in a small series of cases of CL patients with a positive response. However, the dose is not standardized, and, in some cases, injections were used every two weeks or monthly until cure [17–19]. Intralesional antimony therapy has also been used in dogs with CTL caused by *L*. *braziliensis*, mainly in the southern region of Brazil, with good results [20]. However, because self-healing may occur and the cure rates of patients with ATL living in our endemic area are much lower than those observed in other regions, the efficacy of intralesional meglumine antimoniate in dogs with ATL living in the southeast region of Bahia should be determined.

## Material and methods

### Ethics statement

This study followed the recommendation of the Brazilian Federal Law for animal studies. It was approved by the Ethic Committee for Use of Animals in Research (CEUA: 017–2020) of the Instituto Gonçalo Moniz (IGM), Fundação Oswaldo Cruz (FIOCRUZ-BA).

### Case definition and study design

This study is a randomized controlled clinical trial designed to evaluate the efficacy and effectivity of intralesional meglumine antimoniate compared with intralesional sodium chloride solution in dogs with CTL caused by the *L*. *(V*.*) braziliensis*. CTL was defined by the presence of a typical cutaneous or muzzle lesion associated with the documentation of *L*. *braziliensis* DNA detected by PCR. Domiciliary dog participants of the study were identified by an active search of dogs with lesions suggestive of leishmaniasis in the homes of patients with a previous history of ATL and their neighbors living within a 10Km radius of the health post. After explaining the study’s objective to the dogs’ owners, the dogs were immobilized for a physical examination. The area of the lesion was then cleaned with alcohol (70%), and lidocaine was injected into the border of the lesion. The biopsy was performed with a 3mm punch, and the obtained tissue was added to Eppendorf tubes containing RNA later solution (Ambion, Life Technologies, Thermo Fisher Scientific, USA). In addition to tissue biopsy, 5ml of blood was taken from the lateral saphenous vein to detect anti *L*. *braziliensis* antibodies. Dogs were randomized into two groups (http://www.randomization.com). Group one was treated with intralesional Glucantime (Sanofi-Aventis), and group two received intralesional saline. The use of Glucantime or saline was preceded by applying topic lidocaine. A total of 5ml of Glucantime was administered in the four cardinal points. The administration was performed with a needle 13 x 4.0G, and the application was repeated on day 15 and day 30. Animals in the control group received three injections of NaCl solution at 0.9%, in the same way that Glucantime was applied. Physical examination was performed, and pictures were taken on days 0, 30, 60, and 90 after the start of the therapy. Cure was defined as complete healing of the ulcers in the absence of raised borders. When ulcers remained active, or the healing of the lesions occurred in the presence of raised borders on day 90 after initiation of the therapy it was considered failure. Dogs from both groups who failed therapy received intralesional Glucantime in the same dose that was administered in the group test. To determine if the participants in the study remained cured after 90 days, animals were also evaluated 180 days after initiation of therapy.

### Soluble *Leishmania* Antigen (SLA) and Serologic Test

The SLA was obtained from an isolate of *L*. *braziliensis* as previously described by Reed et al., 1986. Antibodies detection to SLA was performed by ELISA as previously described [21,22]. Sera from uninfected dogs living outside an endemic area of *L*. *braziliensis* transmission were used as control. Absorbances higher than the mean plus two standard deviations (SD) of the negative control sera were considered positive.

### Real-time PCR for detection of *L*. *braziliensis* DNA

In order to detect *L*. *braziliensis* DNA, biopsy fragments from the border of the skin or muzzle lesions of infected dogs were stored in the RNA later solution immediately, maintained at room temperature for approximately six hours and storage at 4°C in the laboratory. The DNA was extracted from the tissues using the DNA purification kit (Promega Co., USA), according to the manufacturer’s recommendations and the PCR was performed as previously described [23].

### Statistical analysis

Continuous variables following a normal distribution, as age, are presented as mean and SD and mean differences were analyzed by the Student T test. Continuous variables that did not follow a normal distribution, such as lesion size, are presented as median and interquartile range (IQR). Differences were analyzed by the Mann-Whitney test followed by the post Dunn test. The difference in proportions were analyzed using the Fisher exact test. The GraphPad Prism was used to perform the statistical analysis. The Kaplan-Meier survival curve was performed to compare the healing time proportion in the two groups and the log-rank test was used to compare the curves.

## Results

The demographic and clinical features of the dogs participating in the study according to the type of therapy received are shown in Table 1. The groups did not differ in age, gender, illness duration, or lesion size. The main site of the ulcer was in the scrotal sac detected in nine (56%) dogs treated with Glucantime and 10 dogs (62%) of the control group. Muzzle lesions were observed in two (12.5%) dogs treated with Glucantime and in one (6.25%) of the animals that received NaCl solution. Both the PCR and serologic tests were positive in all participating dogs of both groups. The cure rate on day 90 was higher (P < .0001) in dogs treated with Glucantime than among those who received saline. Dogs were followed monthly up to the endpoint of the study, and we detected that 12 (75%) of the dogs treated with Glucantime were already cured on day 30, and 14 (87,5%) were cured on day 60. Most animals in the group treated with saline remained with active lesions. The two animals of the group test that failed therapy were cured with a second course of intralesional Glucantime. Of the 14 dogs who failed with saline 10 were cured with intralesional Glucantime.

**Table 1:** Demographic Features, Clinical Characteristics and Response to Treatment of Dogs with Canine Tegumentary Leishmaniasis in the Groups Treated with Meglumine Antimoniate or with 0.9% NaCl (placebo).

		Glucantime	Placebo	
		(N = 16)	(N = 16)	*p-value*
**Age (mean and SD) years old**		5.0 ± 3.6	5.7 ± 3.0	>.05
**Male gender (%)**		11 (68.7%)	13 (81.2%)	>.05
**Illness duration in days, mean (SD)**		814.1 ± 643,3	457.2 ± 701.5	>.05
**Lesion size (median and IQR)**		18 (14.5–26.9) mm	23.5 (17–28.3) mm	>.05
**Positive serologic test**		16 (100%)	16 (100%)	>.05
**Cure on day 90**		14/16 (87.5%)	2/16 (12.5%)	**< .0001** ^*^

^*^Fisher exact test

**Fig 1:**
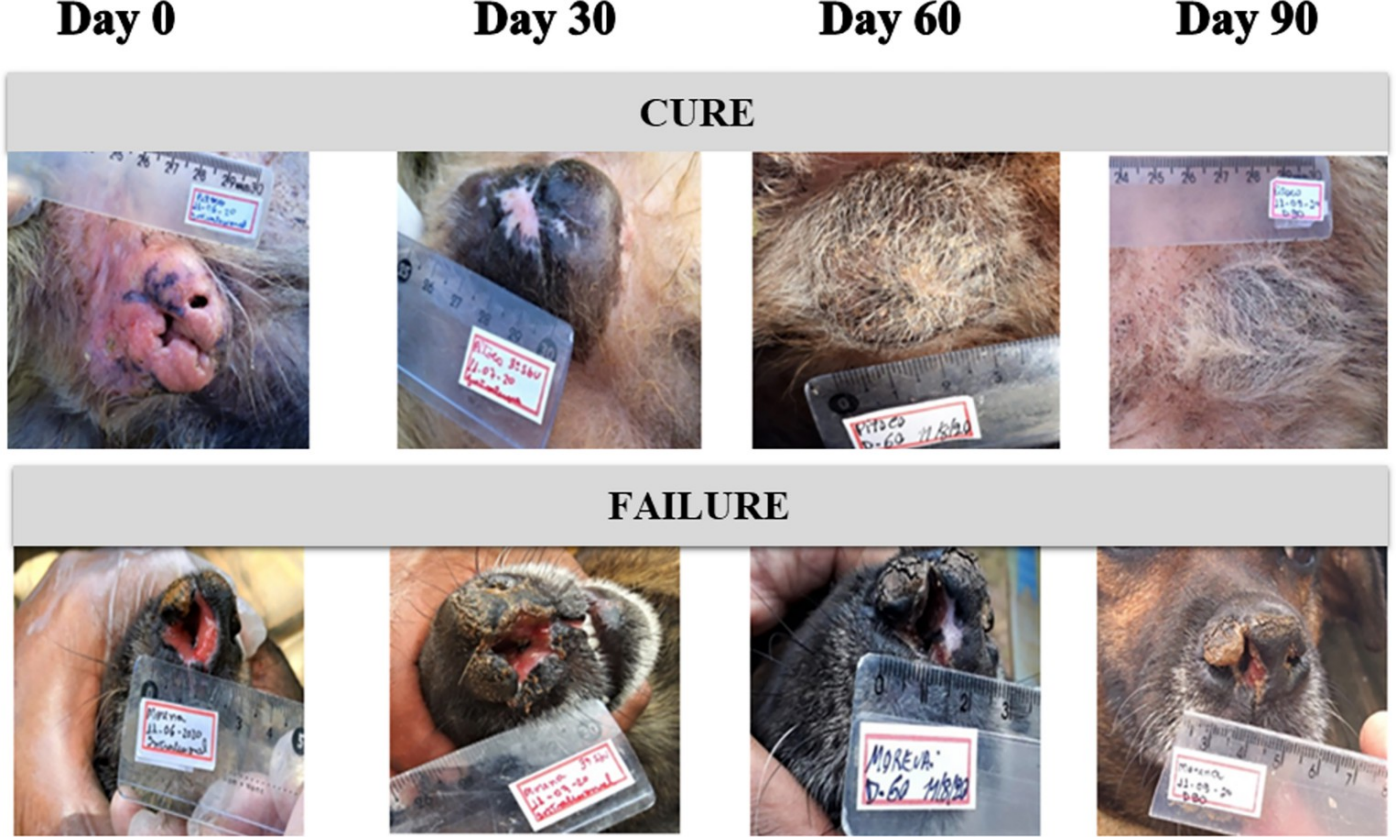
Pictures of two animals on days zero, 30, 60, and 90; “the first, A,” from a dog scrotal sac ulcer treated with Glucantime and “B” from a dog with a muzzle lesion treated with saline. While the dog receiving Glucantime was cured on day 30, the cutaneous lesion remained active until day 90 in the animal treated with placebo.

Fig 1 shows pictures of two animals on days zero, 30, 60, and 90. While the dog receiving Glucantime was cured on day 30 (A), the muzzle lesion remained active until day 90 in the animal treated with 0,9% NaCl solution (B).

### Kaplan-Meier curve

**Fig 2:**
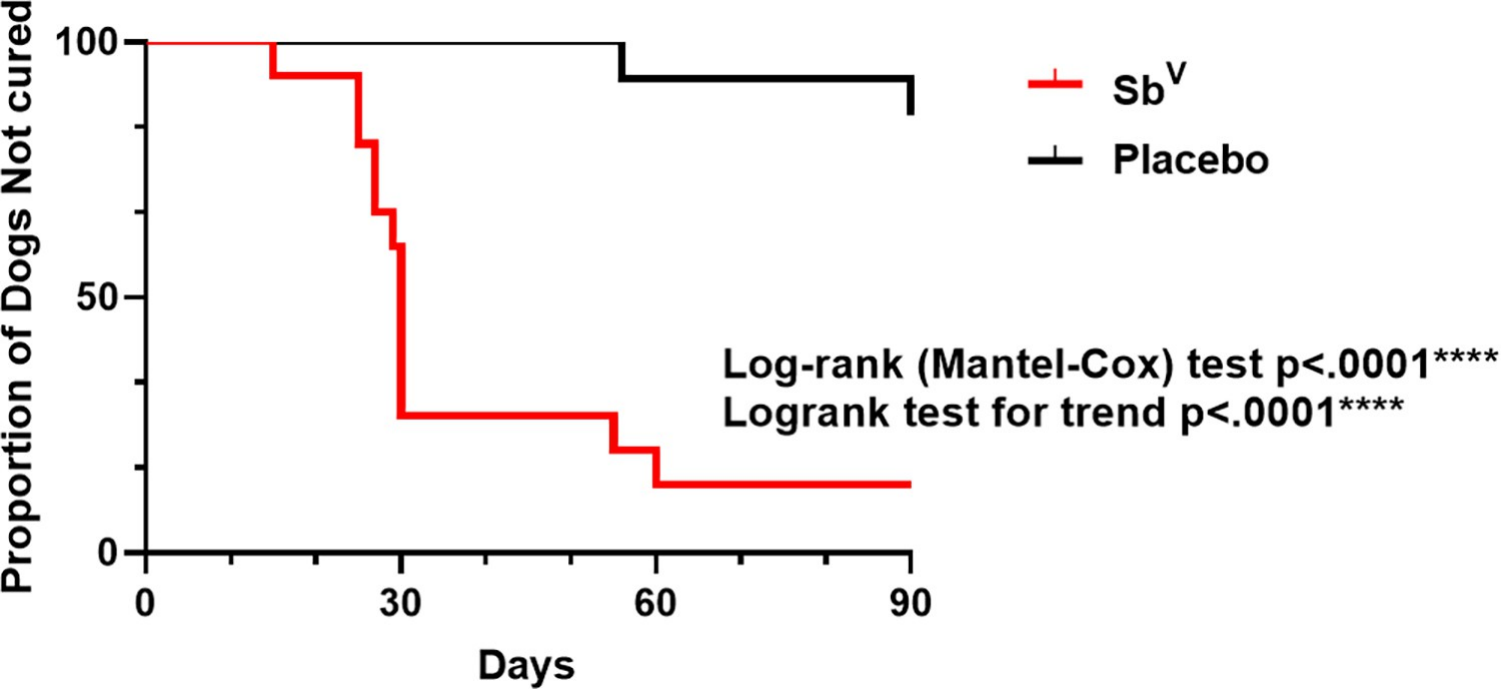
Survival analysis in dogs with CTL. Kaplan-Meier survival analysis of differences in healing time between dogs treated with Glucantime and placebo revealed shorter healing times in dogs treated with Glucantime (P<0.0001, log-rank test).

A Kaplan-Meier curve was performed to compare the proportion of dogs in both groups who achieved cure according to the healing time (Fig 2). The group who received Glucantime reached cure faster than those treated with Placebo (P < .05, log-rank test).

## Discussion

CTL is a relevant public health subject; morbidity is high, the disease remains active for up to a long time, which may contribute to the transmission of the infection to humans. There are, however, no policies for the treatment of CTL. Sick dogs die or are sacrificed. While there is no clear evidence about the role of dogs in *L*. *braziliensis* transmission to humans, dogs are frequently identified with asymptomatic infection or with CTL in areas where human ATL is endemic [4,6].Additionally, a close relationship between the presence of *L*.*braziliensis* infected dogs and the occurrence of CL in humans has been documented [24]. Because there are a few wild and domestic animals with evidence of *L*. *braziliensis* infection, dogs may likely participate in the transmission chain of *L*. *braziliensis*. Moreover, effective therapies for CTL should be searched due to the morbidity observed in dogs infected with *L*. *braziliensis*. In this randomized controlled clinical trial, we documented a higher cure rate of CTL in dogs treated with intralesional meglumine antimoniate than with saline.

All dogs in the study lived in houses, had good nutritional status, and received the recommended vaccines for dogs in Brazil. There were no differences among the groups regarding age, gender, duration of illness, main lesion site, and lesion size. Of note, 24 (75%) of the 32 dogs participating in the trial were males. In humans, CL is also more frequent among males than females, but in such cases, this is explained due to men working more in the forests than women and consequently being more exposed to the infection. However, in Brazil boys have more VL than girls, and *L*. *infantum* is transmitted in the intra or peri domicile area [25,26]. Thus, a relationship between male gender and susceptibility to the development of VL in humans and CL in humans and dogs cannot be ruled out.

In humans infected with *L*. *braziliensis*, lesions occur predominantly in the inferior limbs, which might be explained by the low-level fly [13,27] of the phlebotomies that transmit *L*. *braziliensis*. Here we found that in more than 50% of the dogs, the site of the infection was the scrotal sac, and disease was also observed in the muzzle. As *Leishmania sp* survives in temperatures below 37°C and the scrotal sac and the nose are the coldest areas in the body, the occurrence of diseases in these areas are likely due to the ability of *L*. *braziliensis* to survive in areas with a low temperature in the body.

Dogs with CTL have been treated with different drugs, such as meglumine antimoniate administered intramuscular or intralesional, allopurinol, furazolidone, and domperidone, as well as radio frequency induced heat therapy [28]. In most of these studies, a very small number of animals were used, and all were nonrandomized open trials. In a study with 35 dogs with CTL treated with intramuscular Glucantime, the initial cure was 80.9%, but recurrences at the site of the primary lesion occurred in 42.8% of the dogs [29]. In contrast, in 25 dogs treated with intralesional Glucantime, 86% were cured, and there was no reactivation or appearance of new lesions up to six months after follow-up [20]. Based on the positive response observed in this previous study and the accumulated evidence of positive response to therapy with intralesional Glucantime observed in patients with CL due to *L*. *braziliensis*, we decided to perform this controlled clinical trial. We document that the cure rate of CTL with intralesional Glucantime was higher than observed with saline. Moreover, the healing time in animals treated with intralesional Glucantime was faster than the control group, and 75% of the dogs treated with Glucantime achieved a cure after 30 days of the initiation of the therapy.

In our endemic area, because failure of therapy with intravenous Glucantime may be observed in up to 70% of patients, depending on the clinical forms of the disease and the illness duration [30–32], the high cure rate observed in dogs in the present study was unexpected. The high rate of failure to antimony in this endemic area has been explained by genotypic differences among the parasites in this region. *L*. *braziliensis* in our endemic area is polymorphic and genotypic differences among isolates are associated with the severity of the disease and failure of therapy [33–35]. However, as we found that isolates of *L*. *braziliensis* from dogs living in Corte de Pedra are genotypically similar to the one obtained from patients with CL [4], it is possible that intralesional administration of Glucantime may be associated with better response to therapy than the parenteral administration of the drug.

We recognize this study has some limitations. The sample size was small, and the duration of the illness was almost twice higher in the test group compared to the placebo, although statistical difference had not been documented. The dogs remained during the whole period of the study at home with their owners, and we cannot rule out that other popular therapies were used. However, the differences in cure rate and in the decrease of the healing time of the dogs in the groups, emphasize that intralesional Glucantime might be recommended for treating dogs with CTL caused by *L*. *braziliensis*.
