# Correction: Efficacy of intralesional meglumine antimoniate in the treatment of canine tegumentary leishmaniasis: A Randomized controlled trial

**DOI:** 10.1371/journal.pntd.0011261

**Published:** 2023-04-05

**Authors:** Jamile Lago, Deborah Fraga, Luiz Henrique Guimarães, Tainã Lago, Yuri de Jesus Silva, Ednaldo Lago, Guilherme L. Werneck, Olívia Bacellar, Edgar M. Carvalho

The fifth author’s name is spelled incorrectly. The correct name is: Yuri de Jesus Silva. The correct citation is: Lago J, Fraga D, Guimarães LH, Lago T, Silva YdJ, Lago E, et al. (2023) Efficacy of intralesional meglumine antimoniate in the treatment of canine tegumentary leishmaniasis: A Randomized controlled trial. PLoS Negl Trop Dis 17(2): e0011064. https://doi.org/10.1371/journal.pntd.0011064
